# Economic Evaluation of Boceprevir for the Treatment of Patients with Genotype 1 Chronic Hepatitis C Virus Infection in Hungary

**DOI:** 10.36469/9854

**Published:** 2013-06-05

**Authors:** Raymond Odhiambo, Jagpreet Chhatwal, Shannon Allen Ferrante, Antoine El Khoury, Elamin Elbasha

**Affiliations:** 1 MSD Pharma Hungary Kft., Hungary; 2 Graduate School of Public Health University of Pittsburgh, Pittsburgh, PA, USA; 3 Merck Sharp & Dohme Corp., Whitehouse Station, NJ, USA

**Keywords:** ribavirin, peginterferon, markov model, cost-effectiveness, boceprevir, chronic hepatitis c

## Abstract

**Background:** Recent international, randomized, placebo-controlled clinical trials (SPRINT-2; RESPOND-2) demonstrated that the triple combination of peginterferon (PEG), ribavirin (RBV) and boceprevir (BOC) was more efficacious than the standard dual therapy of PEG and RBV in treatment of patients chronically infected with genotype 1 hepatitis C virus (HCV) infection. The objective of this study was to evaluate the cost-effectiveness of triple therapy in both treatment-naive and treatment-experienced patients in Hungary.

**Methods:** A Markov model was developed to evaluate the long-term clinical benefits and the costeffectiveness of the triple therapy from the Hungarian payer perspective. Model states were fibrosis (F0–F4, defined using METAVIR fibrosis scores), decompensated cirrhosis (DC), hepatocellular carcinoma (HCC), liver transplantation (LT), and liver-related deaths (LD). Efficacy was estimated from SPRINT-2 and RESPOND-2 studies. Disease progression rates and health state utilities used in the model were obtained from published studies. Estimates of probability of liver transplantation and cost were based on an analysis of the Hungarian Sick Fund database. All cost and benefits were discounted at 5% per year.

**Results:** Compared to dual therapy, triple therapy was projected to increase the life expectancy by 0.98 and 2.42 life years and increase the quality-adjusted life years (QALY) by 0.59 and 1.13 in treatment-naive and treatment-experienced patients, respectively. The corresponding incremental cost-effectiveness ratios were HUF7,747,962 (€26,717) and HUF5,888,240 (€20,304) per QALY. The lifetime incidence of severe liver disease events (DC, HCC, LT, LD) were projected to decrease by 45% and 61% in treatment-naïve and treatment-experienced patients treated with triple therapy groups in comparison with PEG-RBV treatment.

**Conclusion:** The addition of boceprevir to standard therapy for the treatment of patients with genotype 1 chronic HCV infection in Hungary is projected to be cost-effective using a commonly used willingness to pay threshold of HUF 8.46 million (3 times gross domestic product per capita).

## BACKGROUND

Chronic hepatitis C virus (HCV) infection is a leading cause of chronic liver disease, liver related deaths, hepatocellular carcinoma (HCC); and the most common indication for liver transplantation.[1,2] According to the World Health Organization (WHO), about 130-170 million individuals are infected with chronic HCV infection worldwide.[3] The burden of HCV is substantial, and the cost of treating HCV infection varies from one country to another.[4,5] In the past decade, awareness about HCV infection has grown among international organizations (e.g. the 'World Hepatitis Day'[6] supported by WHO, European Council), professional organizations, patient organizations, lay press, and governments. In addition, in 2007 hepatitis C has been declared by European Parliament as an urgent public health issue. In Europe, 12 million people are estimated to be living with HCV infection.[7]

The prevalence of HCV infection in Hungary is 0.6 to 0.7%, which corresponds to approximately 60,000 to 70,000 HCV-infected persons.[8] Of the six major HCV genotypes, genotype 1 is the most prevalent (98% in Hungary) and the most difficult to treat.[8] Among patients with chronic HCV infection, at least 20% develop cirrhosis over a period of 20–25 years.[9] The risk of developing end-stage liver disease and/or HCC is also high in these patients. In Hungary, infection with HCV is a major public health problem. Diagnosing, treatment and care of advanced liver disease, as well as liver transplantation represent a challenge for both physicians and society.[10,11] Blood donors have been screened for presence of HCV antibodies since 1992.[11] The majority of current patients were infected by blood transfusion given before 1992. However, introducing screening for blood donors has been shifting the source of new patients derived from high risk populations such as healthcare workers, people with tattoo, piercing and intravenous drug users.[12]

Currently, no vaccine against HCV is available; therefore, effective treatment plays an important role in reducing the burden of the disease. The main objective of HCV therapy is to make the virus undetectable for at least 6 months after the treatment ends, which is known as sustained virology response (SVR). The current standard of care (SOC) for management of HCV infection in patients with chronic liver disease is the combination of pegylated interferon and ribavirin (PEG-IFN/RBV).[10] The efficacy of dual therapy depends on many factors such as genotype, viral load, race, age, fibrosis score and prior treatment.[13-17] Other factor such as obesity may be predictor for decreased efficacy of dual therapy.[18-20] The efficacy of the dual combination therapy is about 40% in treatment naive, genotype 1 patients, and only 22% in treatment-experienced genotype 1 patients.[21,22]

The addition of boceprevir, a new serine protease inhibitor launched in 2011,[23] to combination of PEG-IFN/RBV has shown to significantly increase the SVR rates in the treatment of patients with chronic HCV genotype 1. The Serine Protease Inhibitor Therapy 2 (SPRINT-2) and Retreatment with Serine Protease Inhibitor Boceprevir and PegIntron/Rebetol 2 (RESPOND-2) clinical trials[24,25] demonstrated that the SVR rate achieved by adding boceprevir to PEG-IFN/RBV is 67–68% vs. 40% in treatment-naive patients and 59-66% vs. 21% in treatment-experienced patients.

### Objective

The objective of this study was to project the long-term clinical benefits of the addition of boceprevir (registered by the European Medicines Agency [EMA] in July 2011[26]) to the current standard of care, and estimate its cost-effectiveness in comparison with standard of care in patients with genotype 1 HCV-infection in Hungary.

## METHODS

A decision analytic model was developed to simulate boceprevir-based treatment strategies as approved by EMA and the standard dual therapy. Patients who were not cured progressed to the natural history component of chronic HCV infection, which was modeled as a multi-cohort Markov state-transition model (Figure 1). The model projected the incidence of advanced liver disease (decompensated cirrhosis [DC]), HCC and liver transplantation over lifetime. The structure of the model is similar to other published models for HCV.[27-30] The model estimates the health outcomes and costs of different treatment strategies over lifetime horizon in patient cohorts representative of HCV population in Hungary (Table 1).

**Figure 1.** State-transition Diagram for the Markov Model

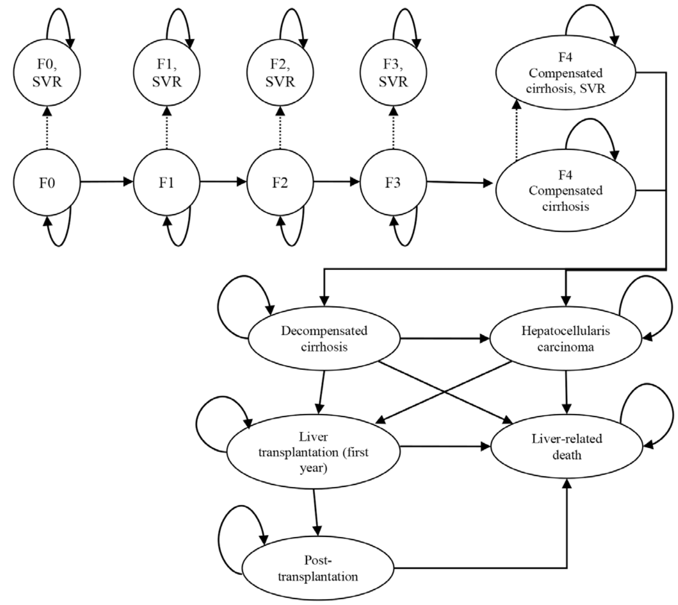

SVR=sustained viral response

**Table 1.** Characteristics of Patients in the Model

| Characteristics | | Value | |
|---|---|---|---|
| **Gender (%)** | | | |
| Male | | 47% | |
| Female | | 53% | |
| **Age** | | 48.1 | |
| **Race** | | | |
| Black | | 0% | 0% |
| Non-black | | 100% | 100% |
| **Baseline METAVIR score*** | | **Treatment-naive** | **Treatment-experienced** |
| | F0 | 4.4% | 4.8% |
| | F1 | 68.9% | 53.3% |
| | F2 | 17.3% | 21.1% |
| | F3 | 4.4% | 7.7% |
| | F4 | 5.0% | 13.1% |

*derived from clinical trials

## Treatment Strategies and Duration

The EMA approved treatment strategies differ according to baseline fibrosis score and prior treatment experience. Separate analyses were conducted for treatment-naive and treatment-experienced patients, with and without cirrhosis.

All patients whether they are treatment-naïve or treatment-experienced, receive PEG-INF/RBV combination therapy during the initial 4-week 'lead-in' period. Subsequently, all patients assigned to control arm are administered 44 weeks of PEG-INF/RBV.

In the 'Boceprevir' (BOC)-arm, the baseline fibrosis score (and response in case of non-cirrhotic, treatment-naïve patients) determines that whether patients receive 24, 32 or 44-week triple combination therapy (PEF-INF/RBV/BOC) after the 4-week 'lead-in'. Both treatment-naïve and treatment-experienced patients who receive 32-week triple combination therapy after initial 'lead-in' period, are administered additional PEG-INF/RBV up to week 48 (Figure 2 and 3).

**Figure 2.** Treatment Strategies in the Model (Treatment-naive Patients)

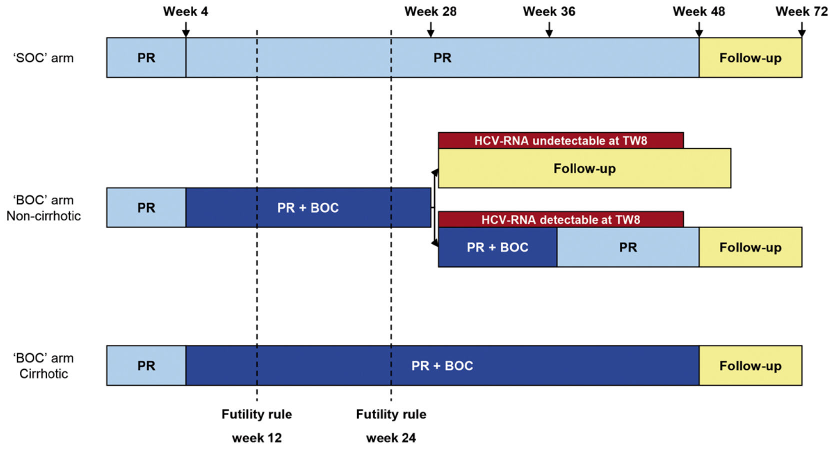

SOC=standard of care (pegylated interferon and ribavirin); BOC=pegylated interferon and ribavirin plus boceprevir;; HCV-RNA=hepatitis C virus ribonucleic acid; TW-treatment week

**Figure 3.** Treatment Strategies in the Model (Treatment-experienced Patients)

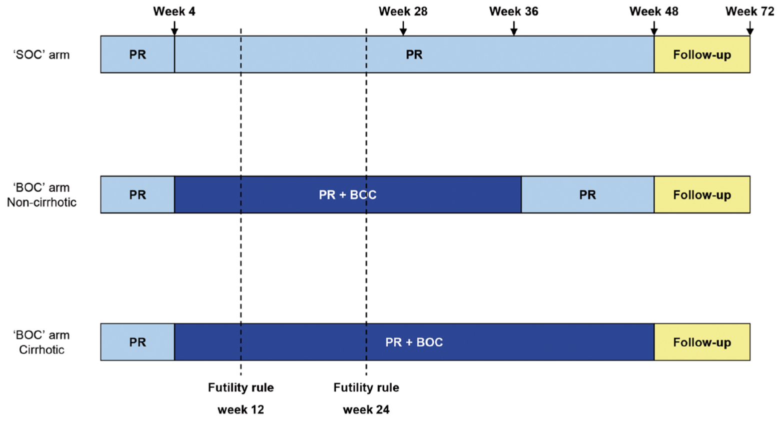

SOC=standard of care (pegylated interferon and ribavirin); BOC=pegylated interferon and ribavirin plus boceprevir;

## Model Structure

For patients who are not cured by treatment, the model simulates the natural course of chronic HCV infection and projects the incidence of advanced liver disease(s) (DC, HCC), and liver transplantation and liver-related deaths over lifetime. The model takes into account a number of health conditions including the severity of chronic HCV infection, which was measured in the trials by the degree of fibrosis using METAVIR scoring system: no fibrosis (F0), portal fibrosis without septa (F1), portal fibrosis with few septa (F2), numerous septa without fibrosis (F3) and compensated cirrhosis (F4). The model assumes that a patient with a given fibrosis score may progress to more severe stages of liver disease, or may remain in that given health state. Patients with compensated cirrhosis are at risk for developing DC and/or HCC. For patients with DC and/or HCC, liver transplantation may be performed. Due to differences in mortality and interventions between periods immediately following transplantation and the following years, liver-transplant state is divided into two stages: "liver transplantation", which lasts for one year, and "Post-liver transplant" state to which a transition is made if a patient is alive at the end of the first year. Patients with DC, HCC, and liver transplantation are at higher risk of mortality compared with the general population. All other patients face the same mortality risk as the general population. We used Excel 2003 (Microsoft Corp., Redmond, Washington) to build the model and to do all calculations.

## Clinical Data

Sustained virological response data as a measure of effectiveness of antiviral therapy were derived from the multicenter, placebo-controlled trials (SPRINT-2[24], RESPOND-2[25]) (Table 2 and Table 3). The target population was patients with genotype 1 chronic HCV infection with or without prior HCV treatment experience. Patient characteristics impact the effectiveness of treatment regimens, the rate of disease progression in patients who do not achieve SVR, and the annual mortality rate. Though SVR rates in SPRINT-2 study were evaluated separately for black and non-black patients, we did not include race in our model because ethnicity along with nationality, mother tongue, religion, chronic illness and disability are regarded as sensitive questions according the Hungarian Population Census. The response to these questions is voluntary.[31] Therefore, no explicit data are available on racial background (black or non-black as defined in the clinical trials[24,25]) in either the total population or HCV-infected population in Hungary. However, it can be inferred from the retrospective analysis of the National Central Statistical Office's database shows that out of "Persons naturalized in Hungary" and "Foreign citizens reside in Hungary"[32] less than 0.5% of total population has (previous) citizenship in a country out of Europe and North America. This also suggests that Hungarian population is mostly white. Therefore, only non-black population was analysed in the non-cirrhotic treatment-naive population.

The clinical trials used METAVIR scoring system to define the severity of hepatitis C disease; however, reporting of fibrosis scores is not mandatory in several cases in Hungary.[8] Since reliable data are not available on the distribution of fibrosis scores in Hungary, we used the distribution of patients' METAVIR scores from the clinical trials.[24,25] To define the average age of patient cohort in the analysis a market research was carried out by an external pharmaceutical market research agency in April 2011. Two hundred twenty patient-reports from 20 centers (out of 30[33]) were involved. Only the reports of patients with finished treatment were included the analysis. The sample was representative of the Hungarian HCV population and included 47% of male and 53% of female patients. The analysis showed that the average age of patients with HCV infection treated is 48.1 years.

**Table 2.** Treatment Outcomes in Treatment-naive Patients

| | Non-cirrhotic* | |
|---|---|---|
| | **SOC** | **BOC** |
| **% experience anemia** | 26.3% | 48.1% |
| **% receive erythropoietin (EPO)/anemia** | 75.3% | 85.2% |
| **Mean duration of anemia (days)** | 104.5 | 88.5 |
| **Mean duartion of EPO use (days)** | 104.9 | 87.5 |
| **% fail futility rule at wk12** | 42.3% | 7.1% |
| **% fail futility rule at wk24** | 9.8% | 11.2% |
| **% SVR*** ** | 36.6% | 68.2% |
| | **Cirrhotic**** | |
| | **SOC** | **BOC** |
| **% experience anemia*** | 23.1% | 54.2% |
| **% receive erythropoietin (EPO)/anemia*** | 100% | 92.3% |
| **Mean duration of anemia (days)*** | 113.3 | 89.8 |
| **Mean duartion of EPO use (days)*** | 236 | 152.8 |
| **% fail futility rule at wk12** | 46.2% | 21.7% |
| **% fail futility rule at wk24** | 14.3% | 22.2% |
| **% SVR**** | 38.5% | 41.7% |

*only non-black **cirrhotic: entire cohort of both black and non-black ***based on EMA label
SOC=standard of care (peginterferon alfa+ribavirin); BOC=peginterferon alfa+ ribavirin + boceprevir; SVR=sustained viral response; EPO=erythropoietin

**Table 3. Treatment Outcomes in the Treatment-experienced Patients**

| | Non-cirrhotic | |
|---|---|---|
| | **SOC** | **BOC** |
| **% experience anemia** | 19.7% | 40.9% |
| **% receive erythropoietin (EPO)/anemia** | 100% | 98.4% |
| **Mean duartion of anemia (days)** | 75.0 | 132.7 |
| **Mean duartion of EPO use (days)** | 66.1 | 137.4 |
| **% fail futility rule at wk12** | 46.9% | 9.0% |
| **% fail futility rule at wk24** | 28.1% | 13.2% |
| **% SVR*** | 24.2% | 64.4% |
| | **Cirrhotic** | |
| | **SOC** | **BOC** |
| **% experience anemia** | 19.7% | 46.7% |
| **% receive erythropoietin (EPO)/anemia** | 100% | 100% |
| **Mean duartion of anemia (days)** | 75.0 | 132.7 |
| **Mean duartion of EPO use (days)** | 66.1 | 131.9 |
| **% fail futility rule at wk12** | 77.8% | 4.8% |
| **% fail futility rule at wk24** | 100% | 5.3% |
| **% SVR*** | 0% | 77.3% |

*based on EMA label; SOC=peginterferon alfa + ribavirin; BOC=peginterferon alfa + ribavirin + boceprevir; SVR=sustained viral response; EPO=erythropoietin

The progression rates describing natural course of chronic HCV infection were derived from Thein *et al.* study.[34] The baseline likelihood of developing HCC in patients with F3 state was estimated from Yoshida *et al.*[35] a retrospective cohort study.[36,37] Annual incidence of HCC increases with development of cirrhosis. An excess risk of HCC and DC from compensated cirrhosis was estimated from Fattovich *et al.*,[38] long-term follow-up study.[39-44] The probability of liver transplantation in advanced stages and mortality rates were derived from the National Health Insurance Fund's database research and analysis, the National Central Office of Statistics and other published data (Table 4).[38,45-50]

**Table 4.** Annual Transition Probabilities

| Parameter | Baseline | Range | Source |
|---|---|---|---|
| **Fibrosis progression** | | | |
| F0 to F1 | 0.117 | 0.104-0.130 | 34 |
| F1 to F2 | 0.085 | 0.075-0.096 | 34 |
| F2 to F3 | 0.120 | 0.109-0.133 | 34 |
| F3 to F4 | 0.116 | 0.104-0.129 | 34 |
| **Fibrosis 3** | | | |
| To HCC* | 0.0095 | 0.00-0.0144 | 35,36,37 |
| **Compensated cirrhosis** | | | |
| To DC | 0.029 | 0.010-0.039 | 35,36,38,39,40,41,42 |
| To HCC | 0.028 | 0.010-0.079 | 35,36,38,39,40,41,42,43,44 |
| **DC** | | | |
| To HCC | 0.068 | 0.030-0.083 | 45 |
| **Probability of liver transplantation** | | | |
| In DC | 0.016 | 0.016 | 46 |
| In HCC | 0.0083 | 0.0083 | 46 |
| **Mortality** | | | |
| Death (all-cause) | Gender- and age specific | | 47 |
| **Liver-related mortality** | | | |
| DC (first year) | 0.182 | 0.065-0.190 | 45 |
| DC (subsequent years) | 0.112 | 0.065-0.190 | 45 |
| HCC | 0.427 | 0.330-0.860 | 38 |
| Liver-transplantation (first year) | 0.166 | 0.060-0.420 | 48 |
| Liver-transplantation (subsequent years) | 0.044 | 0.024-0.110 | 48 |

DC: decompensated cirrhosis; HCC: hepatocellular carcinoma
* Calculated by using Yoshida 1999 [35]

## Health Care Costs and Quality of Life Data

The utility weights associated with health states, treatment and adverse events were obtained from published studies (Table 5).[51-54] The baseline utility weight for the general population was set equal to 1. All health-state utility weights, obtained from Chong *et al.* were normalized to the utility weights of general population. Since anemia was a major adverse event associated with boceprevir-based treatment, we also included the disutility of anemia in the model.

The analysis was conducted from payer's perspective. In Hungary, health services are primarily funded by the state-owned National Health Insurance Fund Administration (NHIFA). The public health services delivered by state- or local government-owned public providers or private players, both contracted with the NHIFA, are registered in the financing database of NHIFA.[55] The cost input data representing real-life resource utilization is based on publicly accessed NHIFA's reports and the results of NHIFA's database research and analyses, as well as using micro-costing methods. Researchers cannot have direct access to the NHIFA's database; however, NHIFA has a regulated service that provides statistical data. A research design was developed involving two researchers and a leading physician in HCV treatment. Both the design and the results of the analyses were validated by two medical experts.

**Table 5.** Parameters of Utility

| Parameter | Baseline | Source |
|---|---|---|
| Utility weights for general population | 1 | |
| **Antiviral (AV) drug therapy-related utility weights** | | |
| PEG-RBV (no side effects) | $(1-0.15)*U_F$ | 52 |
| PEG-RBV-BOC (no side effects) | $(1-0.15)*U_F$ | 52 |
| AV-related anemia | $(1-0.17)*U_F$ | 53 |
| **Post-treatment** | | |
| 'End of Treatment' response | $U_F$ /0.95 | 54 |
| Sustained Virological Response (Cured) | $1.12*U_F$ | 52 |
| **Health state utility weights** | | |
| F0 | 0.78 | 51 |
| F1 | 0.78 | 51 |
| F2 | 0.78 | 51 |
| F3 | 0.78 | 51 |
| Compensated cirrhosis | 0.78 | |
| DC (first year) | 0.74 | 51 |
| DC (subsequent years) | 0.74 | 51 |
| HCC (first year) | 0.55 | 51 |
| HCC (subesquent years) | 0.55 | 51 |
| Liver transplantation (first year) | 0.75 | 51 |
| Liver transplantation (subsequent years) | 0.75 | 51 |

$U_F$ – Baseline fibrosis-related utility
DC: decompensated cirrhosis; HCC: hepatocellular carcinoma

The cost associated with early stages (F0-F3) of the disease was estimated using micro-costing: real-life resource utilization was collected with questionnaires and unit costs derived from public NHIFA's reports were applied. All costs were converted to 2011 Hungarian Forints (Table 6).

Patients who developed treatment-related anemia in the clinical trials[24,25] were managed by reduction of ribavirin dose or administration of erythropoetin (EPO). In Hungary, gradual ribavirin dose reduction is recommended as the most efficient option for management of AV therapy-induced anemia.[56] EPO accessable is neither indicated nor reimbursed in the management of HCV therapy-related anemia in Hungarian setting. Also, a recent study demonstrated that there is no difference in SVR rates achieved in anemic patients receiving boceprevir plus peginterferon-ribavirin using either ribavirin dose reduction or EPO.[57]

In line with the Hungarian therapeutical practice, we assumed that all anemia cases are managed by ribavirin dose reduction. Therefore, the cost of anemia management was set to zero at base case; however, different EPO costs were analysed in the sensitivity analysis.

**Table 6.** Cost Data Used in the Model

| Parameter | Basecase, HUF (€) |
|---|---|
| **Antiviral (AV) drug therapy-related costs (weekly)** | |
| PEG+RBV | 109,311 (€377) |
| Victrelis (boceprevir) | 232,370 (€801) |
| EPO (anemia)* | 110,548 (€381) |
| **Monitoring** | |
| 'Standard of care' | 111,866 (€386) |
| Three medicine combination | 147,192 (€508) |
| **Health state costs (annual)** | |
| F0 | 7,268 (€25) |
| F1 | 25,530 (€88) |
| F2 | 25,530 (€88) |
| F3 | 25,530 (€88) |
| Compensated cirrhosis | 304,617 (€1,050) |
| DC (first year) | 905,011 (€3,121) |
| DC (subsequent years) | 677,955 (€2,338) |
| HCC (first year) | 3,498,735 (€12,065) |
| HCC (subsequent years) | 718,943 (€2,479) |
| Liver transplantation (first year) | 18,309,513 (€63,136) |
| Liver transplantation (subsequent years) | 1,827,296 (€6,301) |
| **Discount rate** | 5% |
| **Time horizon** | Life-time |

*only in sensitivity analysis included; DC=decompensated cirrhosis; HCC=hepatocellular carcinoma; PEG+RBV=peginterferon plus ribavirin

The direct medical cost of HCV infection includes the cost of treatment, HCV health state-related costs, and the cost of liver transplantation. QALYs depend on whether or not the patient is cured, and for uncured patients, the progression of disease and the amount of time spent in each of the HCV disease stages. Both costs and QALYs were discounted at 5%.

### Model Outcomes

We projected the lifetime incidence of complications, total costs, and QALYs associated with each treatment strategy. We also estimated the incremental cost-effectiveness ratios (ICERs) for boceprevir-based regimens compared to treatment with peginterferon and ribavirin. The model estimated the cost-effectiveness of boceprevir-based treatment strategy separately for the treatment-naive and the treatment-experienced patient groups.

There is no official cost-effectiveness threshold in Hungary. Therefore, the WHO-CHOICE[58] guideline around the cost-effectiveness threshold (3 times of gross domestic product (GDP) per capita) was taken into account when interpreting the results. Based on the 2011 National Central Office of Statistics data,[59] 3 times of GDP per capita is 8.46 million Hungarian forint (HUF) (€29,172).

### Sensitivity Analyses

To test the robustness of the model results and the impact of varying parameters on costs and QALYs, both deterministic and probabilistic sensitivity analyses (PSA) were performed. Parameters such as age, transition probabilities, treatment effectiveness, quality of life weights, health care costs, discount rates, implication of EPO cost and mortality in health state of compensated cirrhosis were tested over a plausible range. The base case input data and the lower- and upper bounds of ranges are derived from published data when available (Table 4).

In the base case scenario, both costs and health outcomes were discounted at 5%, as specified in the Hungarian Guideline on preparation health economic analyses . Following the guideline, sensitivity analysis was also performed on the discount rates of costs and health outcomes using the range of 3–6% and 0–6%, respectively. These guidelines are similar to the recommendations of other European national agencies.[61-68]

## RESULTS

### Base-case Analysis

In treatment-naive patients, the boceprevir-based triple combination therapy was projected to increase the life expectancy by 0.98 years and QALY by 0.59 in comparison to treatment with the dual therapy (Table 7). Boceprevir-based therapy costs HUF 4.56 million (€15,707) more than SOC. In treatment-experienced patients, boceprevir-based therapy was projected to increase the expected life by 2.42 life years and QALYs by 1.13, and cost HUF 6.66 million (€22,972) more in comparison to treatment with the dual therapy. Triple therapy resulted in HUF 7,747,962 (€26,717) and HUF 5,888,240 (€20,304) per QALY in the treatment-naive and treatment-experienced patient groups, respectively (Table 7). The relative risk of severe liver disease-related complications such as DC, HCC, liver-related death was projected to decrease over lifetime both in the treatment-naive and the treatment-experienced patients (Table 8).

**Table 7.** Results

| Strategies | LE | Cost (HUF) | QALY | ICER (HUF/QALY) |
|---|---|---|---|---|
| **Treatment-naive patients** | | | | |
| SOC | 25.31 | 4,035,025 (€13,914) | 10.96 | |
| BOC | 26.29 | 8,590,334 (€29,622) | 11.55 | 7,747,962 (€26,717) |
| Δ | 0.98 | 4,555,309 | 0.59 | |
| **Treatment-experienced patients** | | | | |
| SOC | 23.77 | 4,338,644 (€14,961) | 10.35 | |
| BOC | 26.19 | 11,000,397 (€37,932) | 11.48 | 5,888,240 (€20,304) |
| Δ | 2.42 | 6,661,753 | 1.13 | |

SOC=standard of care (peginterferon plus ribavirin)
BOC=boceprevir added to peginterferon plus ribavirin

**Table 8.** Projected lifetime incidence of serious liver complications in the treatment naïve and prevously treated patients (per 1000 patients)

| Patients | Treatment | Liver disease- related complications | | |
|---|---|---|---|---|
| | | DC** | HCC** | Liver-related death |
| **Naive** | SOC* | 100 | 148 | 183 |
| | BOC* | 55 | 80 | 99 |
| | % reduction*** | 45% | 46% | 46% |
| **Previously treated** | SOC* | 153 | 217 | 277 |
| | BOC* | 59 | 86 | 108 |
| | % reduction*** | 61% | 60% | 61% |

*SOC=standard of care (peginterferon plus ribavirin)
BOC=boceprevir added to peginterferon plus ribavirin
**DC=decompensated cirrhosis
HCC=hepatocellular carcinoma
***% Reduction: BOC compared to SOC

## Sensitivity Analyses

The deterministic sensitivity analysis shows that the model results are sensitive to changes in parameters of efficacy (SVR rates), utilities and transition probabilities (Table 9). The ICER obtained from the model exceeded the cost-effectiveness threshold only when assuming the lower value of transition probabilities or the lower incremental health gain (SVR) achieved by boceprevir.

PSA was conducted using 1,000 Monte Carlo simulations and results were presented using cost-effectiveness scatter plots (Figure 4 and Figure 5). The cost-effectiveness acceptability curve (CEAC) showed that the boceprevir-based regimen was cost-effective with a probability of 63% and 93% in treatment-naive and treatment-experienced patients, respectively at a willingness-to-pay value of HUF 8.46 million (Figure 6 and Figure 7).

**Table 9.** The Deterministic Sensitivity Analysis Results (ICER; million HUF)

| Parameter | Tx-naïve, million HUF ('000 €) | Tx-experienced, million HUF ('000 €) |
|---|---|---|
| **Base-case** | 7.75 (26.7) | 5.88 (20.3) |
| **Age (average age of cohort)** | | |
| 35 years old | 5.46 (18.8) | 4.19 (14.4) |
| 43 years old | 6.69 (23.1) | 5.09 (17.6) |
| 53 years old | 9.13 (31.5) | 6.96 (23.9) |
| **Probabilities receiving liver transplantation** | | |
| DC: 0.032; HCC: 0.016 | 7.73 (26.7) | 5.85 (20.2) |
| **EPO cost – 110 548 HUF** | 8.16 (28.1) | 6.49 (22.4) |
| **Discount rate** | | |
| 0% | 2.51 (8.7) | 1.85 (6.4) |
| 3% | 5.27 (18.2) | 3.94 (13.6) |
| Cost:5%; Outcome:0% | 2.92 (10.0) | 2.19 (7.6) |
| **Progression after SVR** | | |
| (DC:0,008; HCC:0,005)* | 7.77 (26.8) | 6.63 (22.8) |
| **Transition probabilities** | | |
| All lower limits | 10.46 (36.1) | 8.64 (29.7) |
| All upper limits | 5.72 (19.7) | 5.03 (17.3) |
| **Compensated cirrhosis → Death (0,0566)**** | 6.38 (21.9) | 4.51 (15.5) |
| **Health state costs** | | |
| -15% | 7.86 (27.1) | 6.02 (20.7) |
| +15% | 7.64 (26.3) | 5.76 (19.8) |
| **Utilities** | | |
| All lower limits | 8.59 (29.6) | 6.47 (22.3) |
| All upper limits | 5.92 (20.4) | 4.72 (16.2) |
| **SVR** | | |
| Low 95% for SOC | 5.97 (20.6) | 5.04 (17.4) |
| High 95% for SOC | 11.27 (38.8) | 9.42 (32.5) |
| Low 95% for BOC | 10.51 (36.2) | 7.72 (26.6) |
| High 95% for BOC | 6.07 (20.9) | 4.70 (16.2) |

SOC: standard of care (peginterferon plus ribavirin)
BOC: triplet therapy (peginterferon –ribavirin plus boceprevir
SVR: sustained virological response
*Source: Cardoso 2010 85
**Calculated by using Flaming 201086

**Figure 4.** The Results of Probabilistic Sensitivity Analysis in Treatment-naive Patients

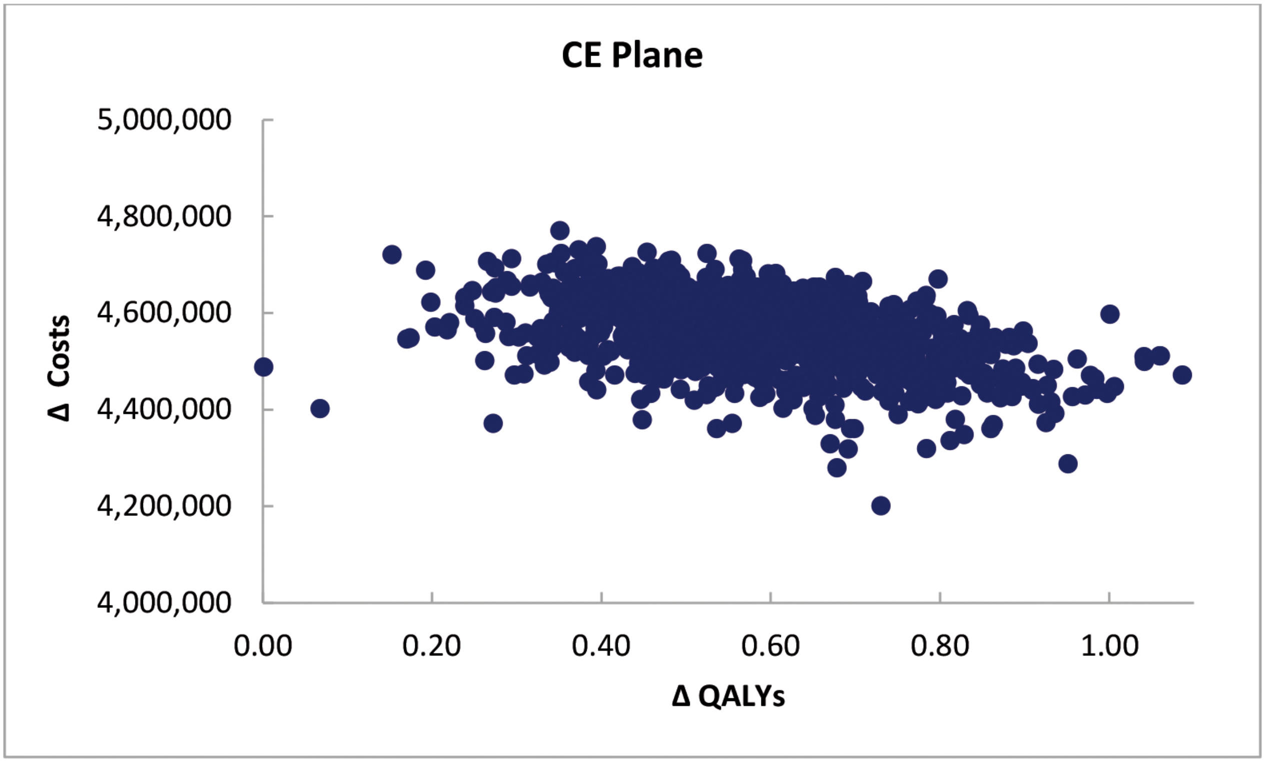

CE=cost effectiveness; QALY=quality-adjusted life year

**Figure 5.** The Results of Probabilistic Sensitivity Analysis in Treatment-experienced Patients

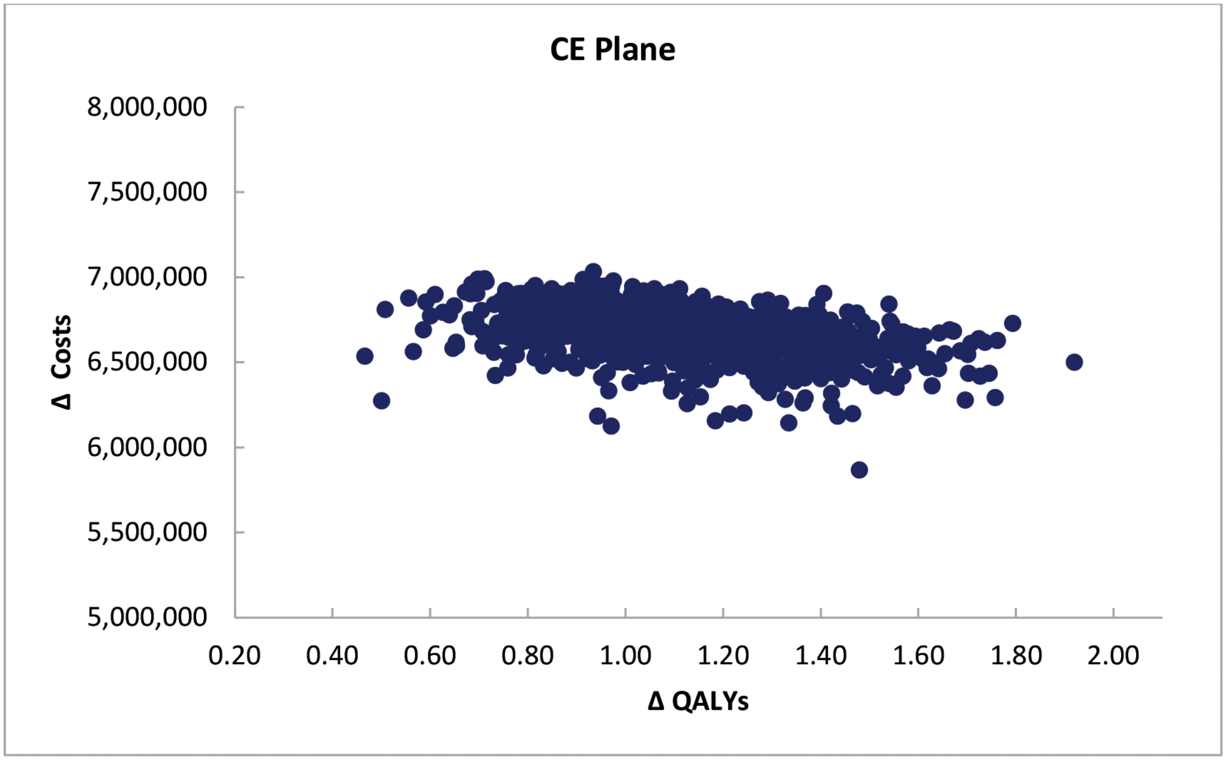

CE=cost effectiveness; QALY=quality-adjusted life year

**Figure 6.** Cost-effectiveness Acceptability Curve in Treatment-naive Patients

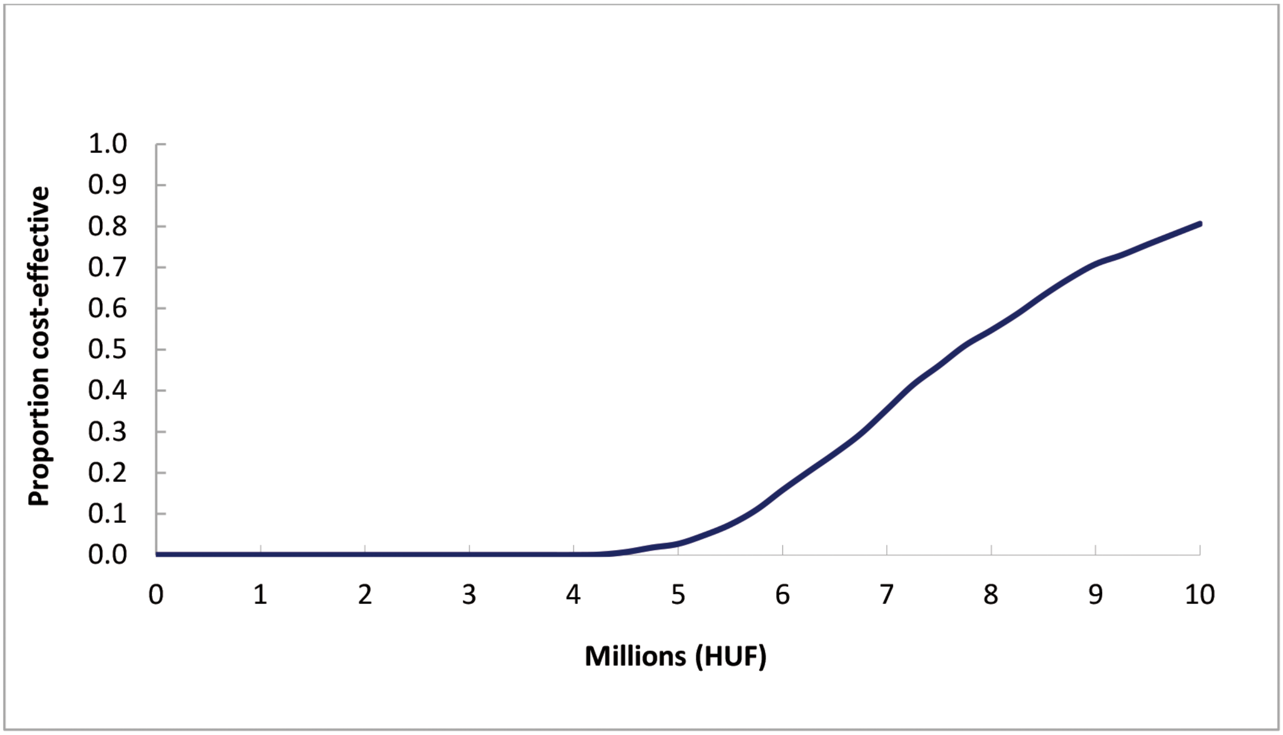

**Figure 7.** Cost-effectiveness Acceptability Curve in Treatment-experienced Patients

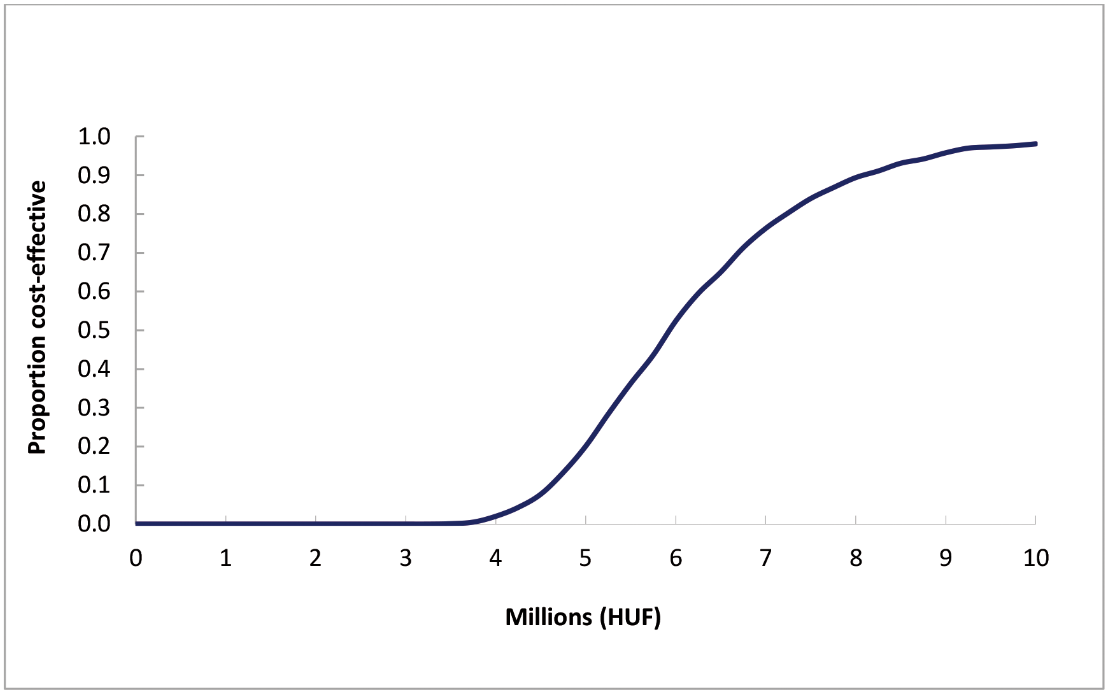

## DISCUSSION

SPRINT-2[24] and the RESPOND-2[25] clinical trial results showed that boceprevir-based triple therapy provides significantly more effective treatment to patients with genotype-1 chronic HCV infection compared with current standard-of-care therapy. At the same time, the scarce resources require the payer to carefully evaluate the economic value of boceprevir when making decision on reimbursements.

The aims of our study were to project lifetime clinical health benefits and estimate the cost-effectiveness of boceprevir-based strategies. The results of our model suggest that boceprevir in addition to peginterferon and ribavirin is projected to be a cost-effective treatment strategy compared with peginterferon plus ribavirin dual combination in both treatment-naive and treatment-experienced patients using a willingness-to-pay threshold of 8.46 million HUF. In the treatment-naive patients, the cost of the boceprevir-based strategies is lower than that in the treatment-experienced patients (HUF 4.56 million (€15,707) vs. HUF 6.66 million (€22,972). This can be explained by the significantly shorter duration of the therapy (28-week vs. 48-week) in non-cirrhotic treatment-naive patients with rapid virologic response. However, the higher additional cost in treatment-experienced patients is counterweighed by the higher incremental health gains (QALYs) in the treatment-experienced patients when compared to the treatment-naive patients. Consequently, boceprevir-based strategy resulted in lower ICER in treatment–experienced patients than in the treatment-naïve patients. The triple therapy is also projected to reduce lifetime incidence of serious liver disease-related complications such as DC, HCC and liver-related death both in the treatment-naïve and treatment-experienced populations. Overall, the results demonstrate that adding boceprevir to peginterferon plus ribavirin provides substantial benefits in terms of value for money in both the treatment-naïve and treatment-experienced patients in the Hungarian setting.

Boceprevir remained cost-effective under a wide range of parameter values in the sensitivity analyses—both in treatment-naive and treatment-experienced patients in the Hungarian setting. The ICER values increased with the reduction of values of transition probabilities and/or incremental efficacy and utilities.

Sensitivity analysis was also performed on the base-case age of HCV-infected population. Currently, the majority of the HCV infected patients in Hungary were infected with blood transfusions prior to 1992.[11] However, the average age of treatment is shifting downward contributing a significant portion of new cases,[8,12] the majority of whom are in their twenties or thirties.[69] Currently the average age of patients with HCV infection is 48.1 years old. However, increasing numbers of intravenous drug users are expected to lower the average age of patients treated with HCV in the future. Therefore, younger cohort was tested in the sensitivity analysis. The results suggest that boceprevir is more beneficial (cost-effective) treatment strategy for the younger cohort of patients infected with HCV compared to the base-case population.

The launch of boceprevir is expected to reduce the burden of liver transplantations because of reduction in the incidence of advanced stage liver diseases. Annually, 40-50 liver transplantations are performed in Hungary.[70-72] Expert estimations indicate 2-3 times more transplantations could be justified based on need.[71,73] In 2012, Hungary joined EuroTransplant, an European non-profit organization responsible for encouraging and allocating organ transplants in its member countries.[74,75] The membership is expected to expand access to liver donors and result in at least two times more liver transplantation. Consequently, the addition of boceprevir to standard therapy may become more favorable because of availability of more liver transplantations.

As with any mathematical model, our model has some assumptions and limitations. First, our model did not include the possibility of spontaneous HCV clearance, which can be observed in patients with mild states (F0 and F1) of HCV. This is similar to SVR called remission. However, likelihood of spontaneous remission in patients with chronic infection is very small;[76-78] therefore, this health state was not included in the model. Second, we assumed that patients who achieved SVR were not at risk for reactivation of HCV infection. Long-term outcomes of several studies[79-84] in HCV infection show that more than 90% of patients who achieved SVR remained virus-free during long-term follow-up.

However, in sensitivity analysis, cirrhotic patients were examined to have an excess risk of developing DC and HCC, even if they achieved SVR.[85] Third, patients with compensated cirrhosis were assumed to have the same mortality risk as the general population. The sensitivity analysis showed that increasing the mortality risk of patients with compensated cirrhosis improves the cost-effectiveness ratio.

## CONCLUSION

The addition of boceprevir to the standard therapy (pegylated interferon plus ribavirin) regimen is projected to substantially reduce the burden of HCV-related liver complications. In addition, the boceprevir-based therapy is projected to be cost-effective when compared to the standard therapy in both treatment-naive and in treatment-experienced patients with chronic HCV infection in Hungary.

## CONFLICT OF INTEREST DECLARATION

Jagpreet Chhatwal, Assistant Professor at the University of Pittsburgh, made a part of his contribution to the model development and manuscript while employed at Merck, Sharp & Dohme Corporation. Jagpreet Chhatwal's effort was supported by the National Center For Advancing Translational Sciences of the National Institutes of Health under Award Number KL2TR000146. The content is solely the responsibility of the authors and does not necessarily represent the official views of the National Institutes of Health. Antoine El Khoury was employed by Merck, Sharp & Dohme Corporation at the time the study was conducted.
